# Independent Variables for Determining the Cumulative Live Birth Rates of Aged Patients with Polycystic Ovary Syndrome or Tubal Factor Infertility: A Retrospective Cohort Study

**DOI:** 10.3389/fendo.2021.728051

**Published:** 2022-01-17

**Authors:** Yichun Guan, Pingping Kong, Zhiying Xiao, Junyan Zhang, Jingfang He, Wenjun Geng, Junfang Yan, Simin Sun, Mingkun Mu, Xiaofang Du, Xingling Wang

**Affiliations:** ^1^ The Reproduction Center, The Third Affiliated Hospital of Zhengzhou University, Zhengzhou, China; ^2^ Training Department, Bothwin Clinical Research Consultants, Redmond, WA, United States

**Keywords:** polycystic ovary syndrome, advanced age, ≥35 years, cumulative live birth rates, tubal factor infertility

## Abstract

**Objective:**

To assess whether women of advanced age (≥35 years) with polycystic ovary syndrome (PCOS) have the same cumulative live birth rate (CLBR) as their age-matched controls with tubal factor infertility and to determine the influencing factors on the CLBRs of aged women.

**Design:**

A retrospective cohort study.

**Setting and Population:**

A total of 160 women of advanced age (≥35 years) with PCOS and 1073 women with tubal factor infertility were included in our study. All patients underwent their first fresh cycles and subsequent frozen cycles within in one year in our centre from 2015 to 2020.

**Methods:**

To determine independent influencing factors on the CLBRs of these aged patients, a multivariable Cox regression model of CLBR according to the transfer cycle type was constructed. *Main outcome measure(s)*: CLBRs.

**Result:**

The Cox regression model of the CLBRs indicated that there was no significant difference between the PCOS group and the tubal infertility group in terms of advanced age (HR, 0.95; 95% CI, 0.71-1.27, *P*=0.732). The CLBR significantly decreased for women of advanced reproductive age up to 37 years of age (HR, 0.46; 95% CI, 0.39-0.56, *P*<0.001). The CLBR increased by 63% when more than ten oocytes were retrieved (HR, 1.63; 95% CI, 1.34-1.98, *P*<0.001). Patients with an AMH level above 32.13pmol/l were likely to have a 72%(HR, 1.72; 95% CI, 1.08-2.73, = 0.023) and 34% (HR, 1.34; 95% CI, 1.07-1.68, P=0.010)improvement in CLBR compared to those with an AMH below 7.85pmol/l and 7.85-32.12pmol/l, respectively.

**Conclusion:**

Despite the higher number of oocytes retrieved in PCOS patients, the reproductive window is not extended for PCOS patients compared with tubal factor infertility patients. Age, AMH and the number of oocytes retrieved play crucial roles in the CLBRs of patients of advanced age (≥35 years).

## 1 Introduction

Polycystic ovary syndrome (PCOS) is a widespread reproductive disorder that encompasses many associated health conditions and impacts various metabolic processes. This condition leads to an increased risk of insulin resistance (IR), type 2 diabetes, obesity, and cardiovascular disease. The aetiology of PCOS remains unclear (1). The three main phenotypic characteristics of this condition are hyperandrogenism, polycystic ovaries, and ovulatory dysfunction (2).

PCOS is the most common cause of menstrual irregularity that leads to infertility. Among all cases comprising couples seeking treatment for infertility, 30% are due to anovulation (3). It is estimated that 90% of anovulation cases are due to PCOS (4). The most common treatments used for ovulation induction are clomiphene citrate (CC) and letrozole (5). For PCOS patients who exhibit CC resistance and letrozole failure, assisted reproductive technology (ART) may play a role in helping them to achieve pregnancy (4, 6). Moreover, *in vitro* fertilization (IVF) and intracytoplasmic sperm injection (ICSI) are the most popular ART treatments.

Traditionally, the success rates of IVF/ICSI have been reported in terms of the number of live births per embryo transfer cycle. With the increasing number of frozen-thawed cycles, it has become essential that we report the outcomes not only of fresh embryo transfers but also of frozen embryo transfers as a complete measure of the success of IVF/ICSI treatment (7). The cumulative live birth rate (CLBR), which includes fresh and subsequent frozen-thawed embryo transfer (FET) cycles, may be one of the most meaningful outcome from the patient’s perspective (8). Women with PCOS have a higher ovarian reserve and number of oocytes retrieved than women with tubal infertility (9). Previous research has shown that the higher the number of oocytes retrieved is, the higher the CLBR will be (8). It seems that women with PCOS should have a higher CLBR. However, with fresh cycles, women with PCOS over the age of 40 have similar clinical pregnancy and live birth rates to those of women with tubal factor infertility (9).

Several studies utilized CLBR of PCOS patients as their primary outcome measure. Nevertheless, some of these studies have taken into account different PCOS phenotypes instead of advanced age, such as hyperandrogenic PCOS phenotypes (10) and female obesity (11). While others focused on the CLBRs of PCOS women who underwent IVM (12) or different controlled ovarian hyperstimulation protocols (13). Samer Tannus et al. studied CLBR in PCOS women over the age of 40 with only a small number of PCOS women included (14). Moreover, few studies have focused on the CLBRs of women of advanced age (≥35 years) with PCOS who are treated by IVF/ICSI. Therefore, we aimed to determine whether aged women with PCOS have the same CLBRs as women with tubal infertility and to characterize the influencing factors on the CLBRs of aged women with infertility.

## 2 Materials and Methods

### 2.1 Patients

This retrospective cohort study included 1233 patients who had undergone IVF/ICSI in our centre between July 2015 and January 2020. Written approval for this study was obtained from the Ethics Committee of The Third Affiliated Hospital of Zhengzhou University.

All patients underwent their first fresh cycles and subsequent frozen cycles within 1 year after oocyte retrieval in our centre and the patient’s age was ≥35 years. According to the cause of infertility, patients were divided into two groups: the PCOS group (160 PCOS patients) and tubal infertility group (1073 patients). PCOS was diagnosed according to criteria from the Rotterdam European Society for Human Reproduction and Embryology/American Society for Reproductive Medicine–Sponsored PCOS Consensus Workshop Group (15). The patients in the tubal infertility group were with or without male factor infertility. The exclusion criteria were as follows: 1) Infertile patients with endometriosis, uterine submucosal fibroids and uterine malformations; 2) infertile patients with endocrine or metabolic diseases; 3) cycles with oocyte donation; or 4) cycles with pre-implantation genetic testing for aneuploidy (PGT-A). Patients who did not have a live birth and had remaining embryos were also excluded.

**Figure 1:**
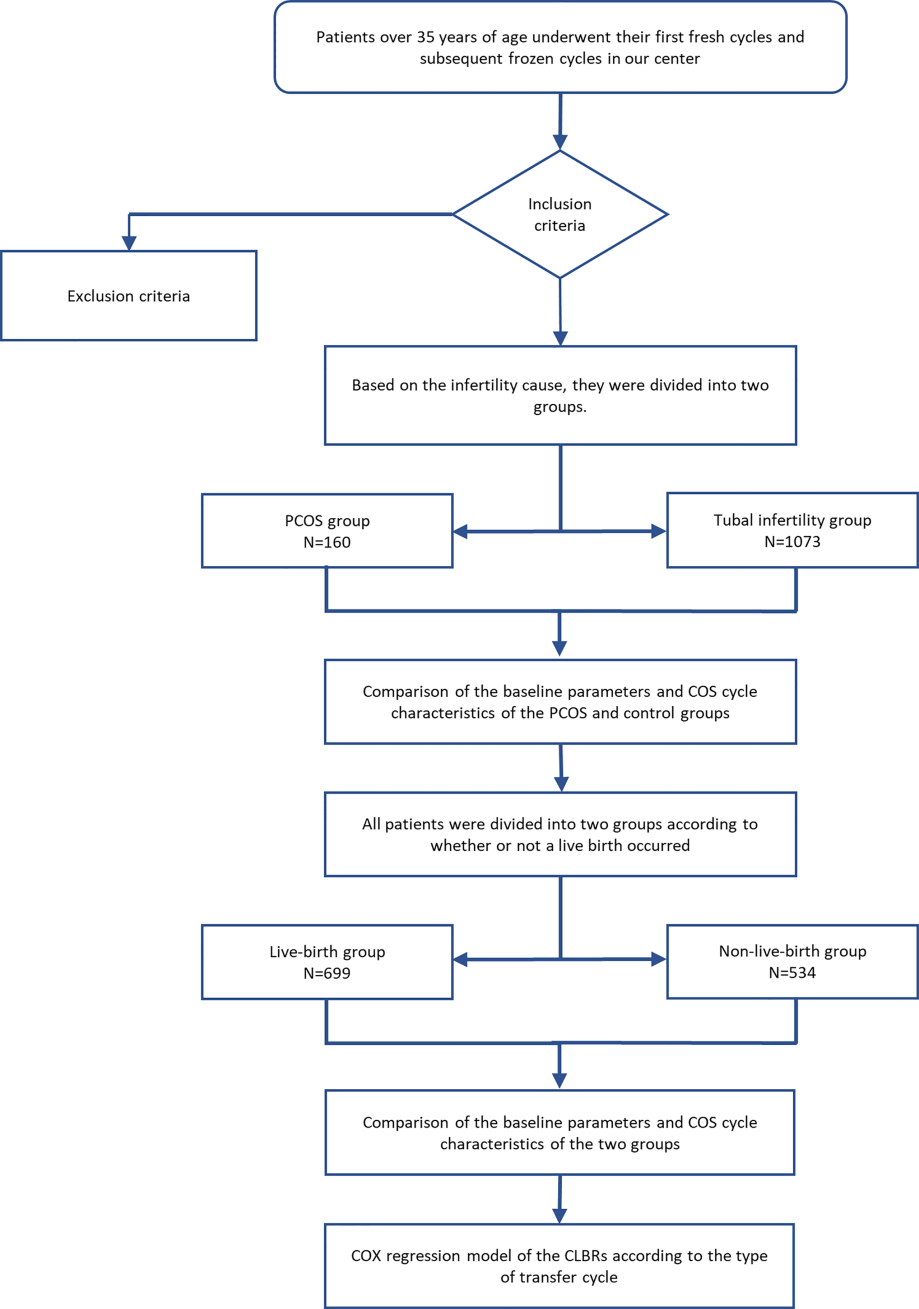
Trial flow chart. An overview of the patient selection and grouping.

The patients’ characteristics, including age, body mass index (BMI), duration of infertility, reproductive endocrine hormone levels, fresh cycle outcomes and CLBRs were evaluated and recorded. The diagnosis of insulin resistance depended on the insulin release test performed by the patient (16). Insulin resistance was considered if the insulin level 1 hour after glucose administration was 5-6 times higher than the fasting insulin level, or if the insulin level 2 hours after glucose administration was 4-5 times higher than the fasting insulin level. An overview of the patient selection and grouping is provided in **Figure 1**. The endpoint of this study was the first live birth of the patient or no remaining embryos for the patient. Any missing data were excluded from the analysis.

### 2.2 Methods

#### 2.2.1 Treatment Protocol

The protocol used for controlled ovarian stimulation (COS) was a long gonadotrophin-releasing hormone (GnRH)-agonist protocol for IVF or ICSI. All patients were treated with recombinant and/or urinary gonadotrophins (Gonal-F, Merck Serono, Germany; or Puregon, Organon, Netherlands; or hMG, Livzon, China) with or without supplementation with recombinant luteinizing hormone (rLH) (Luveris, Merck Serono, Germany). The initial dose ranged from 112.5 to 300 IU per day depending on age, BMI and the results of ovarian reserve tests. During stimulation, the ovarian response was monitored by serial transvaginal ultrasound and serum hormone level measurements.

Ovulation was triggered with recombinant HCG (r-HCG, Ovidrel, Merck Serono, Germany) in all cases, when at least three follicles 18 mm in diameter were observed. Oocytes were retrieved 36-38 h later. Embryo transfer was carried out under ultrasound guidance three or five days after oocyte retrieval. Supernumerary embryos were cryopreserved following vitrification standard protocols.

For luteal phase support, the use of vaginal progesterone gel (Crinone, Merck Serono, Germany: 90 mg every 24 h) or micronized natural progesterone capsules (Utrogestan, Besins, Belgium: 200 mg every 8 h) combined with oral dydrogesterone (Duphaston, Abbott, Netherlands: 10 mg every 12 h) was started on the day of retrieval and continued until the beta-hCG measurement showed that the patient was negative for beta-hCG or until the tenth week of pregnancy.

#### 2.2.2 Endometrial Preparation Protocol

For FET cycles, endometrial preparation for frozen-thawed cycles involved hormonal replacement therapy or ovulation induction cycles. In the hormone replacement cycle, oral oestrogens (Progynova, Bayer, Germany) and micronized progesterone were given with or without pituitary suppression with triptorelin depot 3.75 mg (Diphereline, Epsen, France). In the ovulation induction cycles, patients were administered 2.5 to 5 mg of letrozole daily for five days. If the diameter of the dominant follicle was ≥18-20 mm, ovulation was then stimulated with 10,000 IU of hCG (hCG, Livzon, China) (17).

### 2.3 Outcome Measures

The primary outcome was CLBRs, defined as the delivery of a live born infant (after 28 weeks of gestation) in the fresh or the subsequent frozen-thawed cycles. Only the first delivery was considered in the analysis.

### 2.4 Statistical Analysis

Patients were divided into two groups according to the cause of infertility (PCOS vs tubal infertility). For continuous variables, Student’s t-test was used for data with homogeneous variance, and the Mann–Whitney test was used for data with heterogeneous variance. The χ^2^ test was used for categorical variables. Variables with greater clinical importance or with large variances were selected for further assessment.

The cohort was analyzed using multivariable Cox regression analyses to determine independent risk factors of CLBR. Time was from oocyte retrieval to live birth at risk of CLBR. The end point of time was the time to obtain a live birth or 24 month. Risk factors were cause of infertility, age, BMI, AMH, the number of oocytes retrieved, rLH supplementation, and BMI among others. We adopted the receiver operating characteristic (ROC) curve to find the best point of sensitivity and specificity as the cut off value. In order to application to clinical practice, the nearest integers were adopted. To examine infertility cause, patients were categorized into the PCOS and tubal groups. To examine age and the number of retrieved oocytes patients were categorized into two age groups (age: 35-37 and >37 years, number of retrieved oocytes: ≤10 and >10) according to ROC curve (**Supplementary Figure 1**). Patients in our study were stratified into two groups based on the BMI guidelines for the Chinese population(below 24 kg/m^2^ and above 24 kg/m^2^) (18). Patients were divided into three groups based on AMH values estimated for ovary response(<7.85pmol/l, 7.85-32.13pmol/l, ≥32.13pmol/l) (19–21).The CLBRs after IVF and for certain study factors are presented as the hazard ratio (HR) with the 95% confidence interval (CI). Analyses were performed using Stata 13.1 (Serial number: 401306302851).

## 3 Results

### 3.1 Tubal Factor Infertility and PCOS Groups

As shown in **Table 1**, although the basal testosterone (T) levels were stable across the two groups, a higher BMI (25.43 ± 3.37 *vs* 23.80 ± 2.98, *P*<0.001), higher LH/FSH ratio (1.23 ± 1.01 *vs* 0.67 ± 0.39, *P*<0.001), and longer duration of infertility (5.36 ± 3.88 *vs* 4.30 ± 3.88, *P*=0.001) were observed in the PCOS group than in the tubal factor group. Due to the characteristics of PCOS, the patients of PCOS group had more AFC (23.56 ± 4.98 *vs* 13.40 ± 4.99, *P*<0.001) and higher AMH level (45.07 ± 24.02 *vs* 19.22 ± 11.13, *P*<0.001). In addition, younger age (36.66 ± 2.10 *vs* 37.74 ± 2.55, *P*<0.001), a higher number of oocytes retrieved (15.44 ± 6.71 *vs* 11.35 ± 5.92, *P*<0.001) and a higher rate of rLH supplementation (76.88% *vs* 52.47%, *P*<0.001) were observed in the PCOS group.

**Table 1:** Demographics and IVF/ICSI treatment characteristics of the women with tubal infertility and PCOS.

	PCOS (n = 160)	Tubal infertility (n = 1073)	t	χ^2^	*p*-value
Age (y)	36.66 ± 2.10	37.74 ± 2.55	-5.123		<0.001*
Infertility duration (y)	5.36 ± 3.88	4.30 ± 3.88	3.215		0.001*
BMI (kg/m^2^)	25.43 ± 3.37	23.80 ± 2.98	6.349		<0.001*
Basal FSH (IU/L)	6.47 ± 1.76	7.27 ± 2.26	-4.316		<0.001*
Basal LH (IU/L)	7.56 ± 5.43	4.69 ± 2.54	11.001		<0.001*
LH/FSH	1.23 ± 1.01	0.67 ± 0.39	12.745		<0.001*
Basal T (nmol/l)	0.89 ± 00.44	0.65 ± 0.38	7.296		<0.001*
AFC	23.56 ± 4.98	13.40 ± 4.99	24.029		<0.001*
AMH (pmol/l)	45.07 ± 24.02	19.22 ± 11.13	20.639		<0.001*
Insulin resistance (%)^1^*^1^*	79.61% (82/102)	78.71% (159/202)		0.033	*0.855*
Starting Gn (IU)	189.45 ± 57.88	252.04 ± 99.70	-7.747		<0.001*
Gn duration (day)	13.31 ± 2.70	13.00 ± 2.19	1.656		*0.098*
rLH supplementation (Y/N) (%)	76.88% (123/160)	52.47% (563/1073)		33.600	<0.001*
Number of oocytes retrieved	15.44 ± 6.71	11.35 ± 5.92	8.000		<0.001*
Number of 2PN	10.31 ± 5.76	7.09 ± 4.34	8.339		<0.001*

Values are expressed as percentage (%) or mean and standard deviation (SD). P-value < 0.05 (asterisk) was considered statistically significant. (IVF, in vitro fertilization; ICSI, intracytoplasmic sperm injection; PCOS, Polycystic ovary syndrome; BMI, body mass index; LH, luteinizing hormone; FSH, follicle-stimulating hormone; T, testosterone; Gn, gonadotropin; rLH, recombinant luteinizing hormone).

^1^There were 58 patients in the PCSO group who had no insulin release test, and 871 patients in the tubal infertility group who had no insulin release test. The result of the P value is shown in italics.

Insulin resistance was an important feature of PCOS, but insulin release test was not routinely performed for the aged tubal infertility patients, but only in overweight or obese patients. Therefore, although there is no difference in the incidence of insulin resistance between the PCOS group and the tubal infertility group, the comparison between the two groups is meaningless due to low test rate in tubal infertility group.

### 3.2 Live-Birth Group and the Non-Live-Birth Group

Regarding all transfer cycles (fresh and frozen) outcomes, all patients were divided into two groups depending on whether a live birth was achieved (**Table 2**). Although the BMI and duration of infertility did not differ significantly between the two groups, the basal T levels (0.71 ± 0.42 *vs* 0.64 ± 0.35, *P*=0.001) and LH/FSH ratio (0.78 ± 0.54 *vs* 0.70 ± 0.56, *P*=0.014) were higher in the live-birth group. Nevertheless, younger age (36.90 ± 2.04 *vs* 38.54 ± 2.79, *P*<0.001) was observed in the live-birth group. Moreover, a higher AMH level (26.08 ± 17.44 *vs* 18.64 ± 13.87, *P*<0.001), a higher rate of rLH supplementation (58.80% *vs* 51.50%, *P*=0.011), more number of oocytes retrieved (13.53 ± 6.33 *vs* 9.74 ± 5.27, *P*<0.001) were evident in the live-birth group than in the non-live-birth group.

**Table 2:** Demographics and IVF/ICSI treatment characteristics of the live-birth and the non-live-birth groups of fresh cycles.

	Live-birth group (n = 699)	Non-live-birth group (n = 534)	t	χ^2^	*p*-value
Age (y)	36.90 ± 2.04	38.54 ± 2.79	-11.949		<0.001*
Infertility duration (y)	4.44 ± 3.90	4.43 ± 3.98	0.055		*0.956*
BMI (kg/m^2^)	24.09 ± 3.08	23.91 ± 3.08	0.977		*0.329*
Basal FSH (IU/L)	6.95 ± 2.08	7.44 ± 2.35	-3.868		<0.001*
Basal LH (IU/L)	5.14 ± 3.16	4.96 ± 3.30	0.962		*0.336*
LH/FSH	0.78 ± 0.54	0.70 ± 0.56	2.461		*0.014*
Basal T (nmol/l)	0.71 ± 0.42	0.64 ± 0.35	3.221		*0.001*
AFC	16.00 ± 6.31	13.04 ± 5.23	8.779		<0.001*
AMH (pmol/l)	26.08 ± 17.44	18.64 ± 13.87	7.188		<0.001*
Insulin resistance (%)^1^	82.12% (147/179)	74.60% (94/126)		2522	*0.112*
Starting Gn (IU)	226.73 ± 58.04	66.41 ± 19.26	-7.219		<0.001*
Gn duration (day)	12.97 ± 2.14	13.11 ± 2.42	-1.142		*0.254*
rLH supplementation (Y/N) (%)	58.80% (411/699)	51.50% (275/534)		6.536	0.011*
Number of oocytes retrieved	13.53 ± 6.33	9.74 ± 5.27	11.189		<0.001*
Number of 2PN	9.05 ± 4.85	5.50 ± 3.53	14.279		<0.001*

Values are expressed as percentage (%) or mean and standard deviation (SD). P-value < 0.05 (asterisk) was considered statistically significant. (IVF, in vitro fertilization; ICSI, intracytoplasmic sperm injection; PCOS, Polycystic ovary syndrome; BMI, body mass index; LH, luteinizing hormone; FSH, follicle-stimulating hormone; T, testosterone; Gn, gonadotropin; rLH, recombinant luteinizing hormone).

^1^There were 520 patients in the live-birth group who had no insulin release test, and 408 patients in the non-live-birth group who had no insulin release test. The result of the P value is shown in italics.

### 3.3 Cumulative Live Birth Rates

As shown in **Table 3**, 699 live births were observed. Risk factors were cause of infertility, age, BMI, AMH, the number of oocytes retrieved, rLH supplementation, and BMI among others. Because of low test rate in tubal infertility group, we did not analyze insulin resistance as a confounding factor. The multivariable Cox regression analyses of all transfer cycles (fresh and frozen) concerning timescale showed that the CLBRs of the patients of advanced age with PCOS were not statistically significantly higher than those of the patients with tubal factor infertility (HR, 0.95; 95% CI, 0.71-1.27, *P*=0.732). Compared with that of patients under 37 years old, the CLBR of patients older than 37 years decreased by 54%, which was statistically significant (HR, 0.46; 95% CI, 0.39-0.56, *P*<0.001). The CLBR increased by 63% when more than ten oocytes were retrieved (HR, 1.63; 95% CI, 1.34-1.98, *P*<0.001). Patients with an AMH level above 32.13pmol/l were likely to have a 72%(HR, 1.72; 95% CI, 1.08-2.73, = 0.023) and 34% (HR, 1.34; 95% CI, 1.07-1.68, *P*=0.010)improvement in CLBR compared to those with an AMH below 7.85pmol/l and 7.85-32.12pmol/l, respectively.

**Table 3:** Multivariable Cox regression analyses of CLBRs.

Independent covariates	HR	95% CI	*p-value*
**PCOS vs tubal**	0.95	0.71-1.27	*0.732*
**Age (above 37 vs below 37 years)**	0.47	0.39-0.56	<0.001*
**AMH (pmol/l)**			
**AMH (7.85-32.13 vs below 7.85)**	1.29	0.85-1.96	*0.229*
**AMH (above 32.13 vs below 7.85)**	1.72	1.08-2.73	0.023*
**AMH (above 32.13 vs 7.85-32.13)**	1.34	1.07-1.68	0.010*
**BMI (above 24 vs below 24)**	1.11	0.97-1.01	*0.403*
**Basal T (above 1.70vs below 1.70)**	1.14	0.68-1.92	*0.625*
**Number of oocytes retrieved** **(above 10 vs below 10)**	1.63	1.34-1.98	<0.001*
**rLH supplementation vs no rLH**	1.01	0.85-1.22	*0.686*

P-value < 0.05 (asterisk) was considered statistically significant. (CLBRs, cumulative live birth rates; PCOS, Polycystic ovary syndrome; BMI, body mass index; LH, luteinizing hormone; T, testosterone; rLH, recombinant luteinizing hormone; HR, hazard ratio; CI, confidence interval). The result of the P value is shown in italics.

## 4 Discussion

According to our multivariable Cox regression analyses, age, number of oocytes retrieved and AMH play crucial roles in the CLBRs of patients of advanced age (≥35 years). Despite the higher number of oocytes retrieved in PCOS patients, the reproductive window is not extended in patients of advanced age with PCOS compared to those with tubal factor infertility.

Over recent decades, IVF/ICSI protocols have continued to evolve to improve outcomes. However, there is still a linear decline in the success rate of ART with patient age. In general, advanced age is defined as ≥35 years, and oocyte depletion of the female follicular pool continues from the embryonic stage. After age 35, and especially after age 37, follicle numbers decline bi-exponentially rather than as a simple exponential function of age (22). A previous study also found that the CLBR was age dependent and declined from over 50% for women who were ≤37 years old to 34.1% for women who were 38–40 years old to 17.7% for women who were 41–42 years old (23). Our study is in complete agreement with the study above, demonstrating that compared with that of women younger than 37 years, the CLBR of women over 37 years old significantly decreased by 54%.

Additionally, ageing may also affect the CLBR by affecting oocyte quality. Mitochondria play a central role in follicular atresia and can be the main target of ooplasmic factors that determine oocyte quality. Age-related mitochondrial DNA (mtDNA) instability, which leads to the accumulation of mtDNA mutations in the oocyte, may play a key role in the deterioration of oocyte quality and the risk of transmitting mitochondrial abnormalities to the offspring (24). Previous studies have shown that the incidence of oocyte aneuploidy increases with age (25, 26). Embryo quality may be the main factor affecting the live birth rate after elective single-embryo transfer in fresh stimulation cycles (27).

In addition to age, the number of retrieved oocytes is an important factor in the CLBR. An increased number of oocytes retrieved improves the pregnancy rates of women undergoing IVF/ICSI, not only by increasing the number of available embryos but also by allowing extended embryo culture and enabling the selection of the best-quality embryo for transfer (28). Furthermore, after the fresh embryo transfer, there are more opportunities for FET, which may increase the CLBR. Our study demonstrated that the CLBR increased by 63% when more than ten oocytes were retrieved. This is in agreement with a multivariate logistic regression analysis showing that the number of oocytes retrieved was an independent predictive factor (*P* < 0.001) for the CLBR after adjustments for fertilization rate, age, day of fresh embryo transfer and insemination method (29).

Many factors may directly or indirectly impair the competence of maturing oocytes, resulting in a lower pregnancy rate for patients with PCOS. The identified extra-ovarian factors include hyperandrogenaemia and hyperinsulinaemia, while intra-ovarian factors comprise members of the epidermal, fibroblast, insulin-like and neurotrophin families of growth factors, as well as cytokines. Any abnormality may negatively affect the granulosa cell-oocyte interaction, oocyte maturation and potential embryonic developmental competence, which contribute to unsuccessful outcomes for PCOS patients undergoing assisted reproduction (30). For PCOS patients, abnormal metabolism and insulin resistance have adverse effects on ovarian response, embryo quality and pregnancy outcome. Women with PCOS were at an increased risk of miscarrying a chromosomally aberrant embryo/fetus compared with non-PCOS controls during ART (31). While Li et al. (32) found that among aged women(≥35 years), the PCOS patients exhibited a higher CLBR than the non-PCOS patients, which is not consistent with our outcome. Whether PCOS is an influencing factor in aged patients, further clinical studies on larger sample sizes and mechanism studies are needed.

AMH is not only an important predictor of ovarian response, but also may be associated with live birth (33). The correlation between AMH and pregnancy outcomes in IVF/ICSI patients is controversial. Steiner et al. (34) found that basic AMH level and FSH level had no correlation with cumulative pregnancy rate, and AMH or FSH level could not predict pregnancy outcome of patients. But they studied patients who were trying to conceive by 6 and 12 cycles of attempt instead of IVF/ICSI. Zhang et al. (35) found no relationship between different AMH levels and clinical pregnancy rate and live birth rate through retrospective data, but they did not focus on aged patients and PCOS patients. However, a meta-analysis found that AMH was positively correlated with live birth (36). PARK et al. (37)found that AMH levels were predictive of clinical pregnancy in infertility patients over 40 years of age. This is consistent with our results the higher AMH is, the higher CLBR is in the aged patients.

We also investigated no influence of rLH supplementation on the CLBRs of women of advanced age. With ageing, the number of LH receptors and LH receptor sensitivity decrease due to paracrine deficiency (38). In ovulation cycles, LH is essential for steroid production and the development of follicles (39). It has also been proposed that the endogenous LH level increases with age and that the LH concentration remains high after pituitary downregulation (40). Whether it is necessary to supply rLH during COS remains unclear. However, Schwarze et al. (41) found that there was no association between supplementation with LH and an increased mean number of inseminated metaphase-II oocytes, an increase in the delivery rate, or changes in the miscarriage rate, and the authors suggested that LH supplementation had little impact on the outcome of ART. This is consistent with our study.

To the best of our knowledge, this is one of the first study to determine the CLBRs of women of advanced age with PCOS. A major strength of the current study is its attempt to explore the CLBRs of women of advanced age with PCOS. PCOS is one of the most prevalent endocrine disorders. The reproductive lifespan of women with PCOS may be, on average, two years longer than that of normo-ovulatory women (42). Compared with non-PCOS patients, PCOS patients show a slow decline in ovarian reserve function and delayed physiological ageing of their ovarian follicles (43). Therefore, it seems that the PCOS group would have a higher CLBR.

However, the available data show the opposite. Despite the higher oocyte yield in all age groups of women with PCOS, patients over age 40 with PCOS who underwent fresh cycles had similar clinical pregnancy and live birth rates to those of the women with tubal factor infertility who also underwent fresh cycles (9). These findings suggest that the reproductive window is not extended by PCOS as patients age and that infertile patients should be treated promptly, despite indicators of high ovarian reserve (9). Fertility in patients with PCOS is maintained until the age of 38 years with the use of IVF. Afterward, the pregnancy rate decreases, although the number of oocytes retrieved by IVF remains stable (44). We demonstrated that the CLBR of PCOS patients was comparable to that of the tubal factor infertility patients of advanced age, which may not be consistent with li et al. (32), but we adjusted for more critical confounders in our analysis.

This study expands on previous research, but it has several limitations, most of which are associated with the retrospective study design. First, the compared groups may have differed in terms of several baseline characteristics. Nevertheless, the effects of these differences would be minimal given that these factors were analysed in multivariable Cox regression analyses. Second, due to the incompleteness of the original data, more detailed characterization of presenting features of the PCOS patients that could potentially affect the pregnancy outcome were not included in the analysis. Finally, the live birth rate is closely related to the rate of aneuploid embryos assessed by PGT-A and mitochondrial DNA copy number tested with next generation sequencing (NGS). We did not review the data of aneuploid embryos and mitoscoreo at admission. Whether or not women of advanced age with PCOS have more aneuploid embryos than those with tubal infertility should be assessed.

## 5 Conclusion

Even though their ovarian reserve is high, women of advanced age with PCOS should be given more attention with the help of ART. Despite the higher number of oocytes retrieved, the reproductive window is not extended for patients with PCOS compared with those with tubal factor infertility. Age, AMH and the number of oocytes retrieved play crucial roles in the CLBRs of patients of advanced age (≥35 years).

## Data Availability Statement

The raw data supporting the conclusions of this article will be made available by the authors, without undue reservation.

## Ethics Statement

Written approval for this study was obtained from the Ethics Committee of The Third Affiliated Hospital of Zhengzhou University.

## Author Contributions

YG developed the conceptual framework and research protocol for the study. PK, ZX and WG conducted the data collection. JZ and JH carried out the statistical analysis. JY, SS, MM, and XW interpreted the data. XD edited the flowchart and tables. PK and ZX drafted the manuscript. YG made major revisions. All authors approved the final version of the manuscript.

## Conflict of Interest

Authors JZ and JH were employed by Bothwin Clinical Research Consultants.

The remaining authors declare that the research was conducted in the absence of any commercial or financial relationships that could be construed as a potential conflict of interest.

## Publisher’s Note

All claims expressed in this article are solely those of the authors and do not necessarily represent those of their affiliated organizations, or those of the publisher, the editors and the reviewers. Any product that may be evaluated in this article, or claim that may be made by its manufacturer, is not guaranteed or endorsed by the publisher.
