# Supplementary material for: Independent Variables for Determining the Cumulative Live Birth Rates of Aged Patients with Polycystic Ovary Syndrome or Tubal Factor Infertility: A Retrospective Cohort Study
Source: Front Endocrinol (Lausanne). 2022 Jan 17;12:728051. doi: 10.3389/fendo.2021.728051 (PMC8803204; doi:10.3389/fendo.2021.728051)
Supplement: Supplementary file 1 [file DataSheet_1.docx]

(a) *

(b)*

Supplementary Figure 1. Receiver operating characteristic (ROC) curves for age and number of oocytes retrieved in Cumulative live birth rates (CLBRs).

* Area under the curve (AUC) significantly larger than 0.5.
